# Anki Tagger: A Generative AI Tool for Aligning Third-Party Resources to Preclinical Curriculum

**DOI:** 10.2196/48780

**Published:** 2023-09-20

**Authors:** Tricia Pendergrast, Zachary Chalmers

**Affiliations:** 1 Department of Anesthesiology University of Michigan Medicine Ann Arbor, MI United States; 2 Northwestern University Feinberg School of Medicine Chicago, IL United States

**Keywords:** ChatGPT, undergraduate medical education, large language models, Anki, flashcards, artificial intelligence, AI

## Abstract

Using large language models, we developed a method to efficiently query existing flashcard libraries and select those most relevant to an individual's medical school curricula.

## Introduction

ChatGPT is a natural language processing tool that uses deep learning to generate responses to questions from human users [1]. ChatGPT has many possible applications in health care and medical education [2].

Medical students complete much of their preclinical didactic learning outside of the classroom, with the assistance of third-party resources such as Anki flashcard decks, instead of traditional lectures [3]. Anki flashcard decks use the principle of spaced repetition to improve memorization [4,5]. Medical students found Anki flashcards produced for their specific curriculum helpful and believed that these flashcards reduced anxiety. However, most medical students use open-sourced flashcards available online [6]. These decks are maintained by medical students who collaborate using the social media platform Reddit (/r/medicalschoolanki) [7] and through a subscription-based web application that facilitates crowdsourced peer review of flashcard content [8]. Medical students work together to address errors in the flashcards and update them as needed.

Use of crowdsourced flashcard decks eliminates the investment of time required upfront to produce flashcards for each lecture, but these flashcards are not specific to the user’s medical school curriculum [4]. A mechanism to match existing flashcards, created and vetted by medical students within the Reddit and AnkiHub communities, to the learning goals of didactic lectures delivered by medical school faculty members would be less time-intensive for faculty and students. In this research letter, we describe a novel method to efficiently select relevant flashcards from existing Anki decks and associate those cards with individual lectures within the user’s medical school curriculum.

## Methods

**Figure 1:**
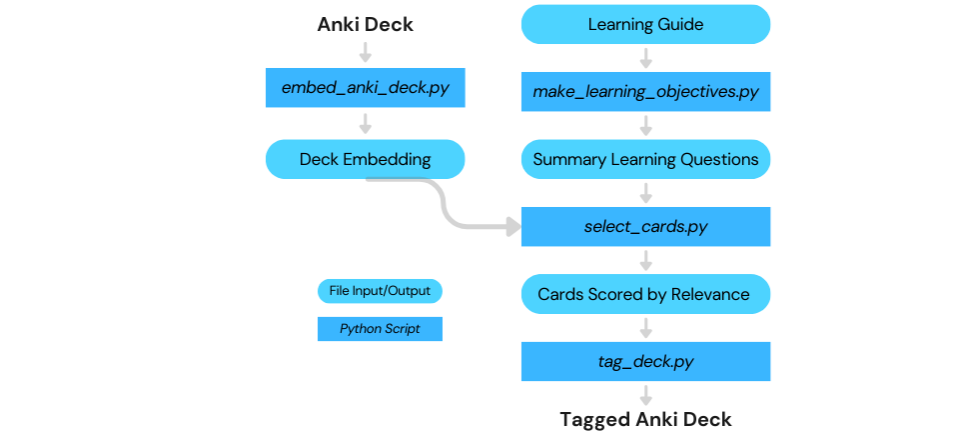
Workflow schematic.

There are 4 core steps in the workflow (Figure 1)*.* The cards of a target Anki deck are embedded in a large language model (LLM). The gpt-3.5-turbo-16k model summarizes the learning guide into a set of comprehensive learning questions. Cards are presorted for their relevance to the learning question, using the LLM deck embedding, and then gpt-3.5-turbo scores the relevance of these cards to the learning question*,* which continues until a user-defined query limit for the learning question has been reached. Finally, cards are tagged in the original Anki file, stratified into “highly relevant,” “somewhat relevant,” or “minimally relevant” categories. Technical documentation and scripts are deposited in GitHub [9].

## Results

**Figure 2:**
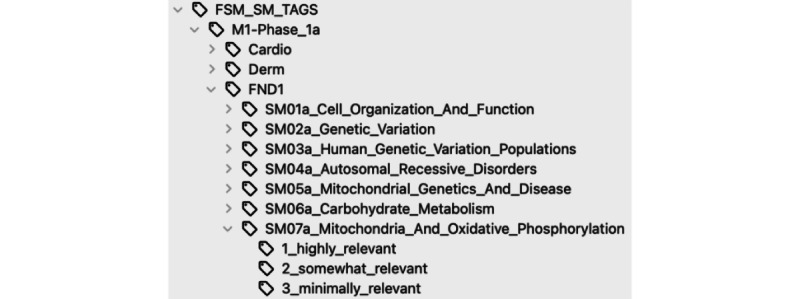
Hierarchical tag structure.

Using the method described above, we selected flashcards from the AnKing flashcard deck that contained 35,152 flashcards and tagged them to our institution’s preclinical curriculum (Figure 2) [8]. We obtained a total of 465 science of medicine lecture guides spanning the 15 system-based modules at Feinberg School of Medicine for the 2022-2023 academic year. For each lecture guide, an average of 13 (range 5-34) summary learning questions were generated by our algorithm. For example, a lecture on central nervous system cancers, might include the following questions: “How do we diagnose and treat gliomas?” and “What genetic syndromes are associated with benign and malignant tumors in the brain?” After generating 4918 unique learning questions, the selection algorithm yielded a total of 21,400 flashcards from the AnKing deck, of which 16,113 were designated as highly relevant to a learning question. On average, 88 (range 11-221) flashcards were selected per lecture. Upon inspection of a sample of lectures, the quality of selections was considered high, with >90% of cards appearing highly relevant. The process developed is highly scalable, with individual lecture guides processed in minutes at minimal computational cost.

## Discussion

It is up to medical schools to decide how to adapt to a status quo increasingly defined by student-driven medical education. One possibility is for medical schools to align the student-driven curriculum with the instructor-led curriculum and consider the incorporation of vetted, third-party resources, such as Anki, into didactic learning [3].

Using large language models, we developed a method to efficiently query flashcards in existing widely used libraries and select those most relevant to an individual's medical school curricula. The feasibility of implementing a ChatGPT flashcard generation into pre-clerkship medical school curricula has not been evaluated and is an area of future study, with algorithmic fine-tuning and prompt optimization likely to further increase the specificity of selections Subsequently, a comparison of medical students’ satisfaction with self-made Anki flashcards compared to ChatGPT-tagged Anki flashcard decks should be conducted.

## Abbreviations

LLM: large language model

## References

[1] Gilson A, Safranek CW, Huang T, Socrates V, Chi L, Taylor RA, Chartash D (2023). How does ChatGPT perform on the United States Medical Licensing Examination? the implications of large language models for medical education and knowledge assessment. JMIR Med Educ.

[2] Ayoub NF, Lee Y, Grimm D, Balakrishnan K (2023). Comparison between ChatGPT and Google Search as sources of postoperative patient instructions. JAMA Otolaryngol Head Neck Surg.

[3] Wu JH, Gruppuso PA, Adashi EY (2021). The self-directed medical student curriculum. JAMA.

[4] Wothe JK, Wanberg LJ, Hohle RD, Sakher AA, Bosacker LE, Khan F, Olson AP, Satin DJ (2023). Academic and wellness outcomes associated with use of Anki spaced repetition software in medical school. J Med Educ Curric Dev.

[5] Jape D, Zhou J, Bullock S (2022). A spaced-repetition approach to enhance medical student learning and engagement in medical pharmacology. BMC Med Educ.

[6] Rana T, Laoteppitaks C, Zhang G, Troutman G, Chandra S (2020). An investigation of Anki Flashcards as a study tool among first year medical students learning anatomy. The FASEB Journal.

[7] Medical School Anki. Reddit.

[8] AnkiHub.

[9] zachalmers - Anki_Tagger. GitHub.

